# The association between family cohesion and disability following blunt trauma: findings from a level-I trauma center in Saudi Arabia

**DOI:** 10.1186/s40621-020-00271-0

**Published:** 2020-08-10

**Authors:** Sarah Mohammed Almarwani, Leen Omar Hijazi, Modhi Abdullah Alamer, Jury Muhanad Alnwaiser, Reem Abdullah Aldakheel, Khalid Alsheikh, Ibrahim Albabtain, Suliman Alghnam

**Affiliations:** 1 King Saud bin Abdulaziz University for Health Sciences, Ministry of National Guard–Health Affairs (NGHA), Riyadh, Saudi Arabia; 2 Department of Orthopedics, Ministry of National Guard-Health Affairs, Riyadh, Saudi Arabia; 3 College of Medicine, King Saud bin Abdulaziz University for Health Sciences, Riyadh, Saudi Arabia; 4 Department of Surgery-Hospital-NGHA, King Abdulaziz Medical City, Riyadh, Saudi Arabia; 5 Population Health Section- King Abdullah International Medical Research Centre (KAIMRC), King Saud Bin Abdulaziz University for Health Sciences (KSAU-HS), Riyadh, 11426 Saudi Arabia

**Keywords:** Saudi Arabia, Trauma, Family cohesion, Disability

## Abstract

**Background:**

Injuries pose a significant burden on population health of Saudi Arabia. Even in nonfatal injuries, the burden varies from temporary to permanent disabilities. Health outcomes following injuries can vary, and predictors of recovery from disability are not well understood. In the Kingdom, family values and cohesion can differ from other countries due to several factors, including religious beliefs and cultural traditions. Learning about predictors of injury recovery can improve prevention as well as planning for rehabilitation programs. Therefore, the study aims to evaluate the association between family cohesion and recovery following blunt injuries.

**Methods:**

This prospective study included 249 patients who were hospitalized for at least 1 day following blunt trauma in King Abdulaziz Medical City, Riyadh. Adult patients were interviewed twice: initially during admission, and a second interview via the phone 3 months after discharge. Baseline information included: demographics, injury characteristics, the five dimensions EQ-5D and family support scale. The follow-up interview captured only EQ-5D. Suboptimal family cohesion was defined as any issue with the relationship with parents, spouse, or siblings. Any disability was defined as a reported limitation in one or more domains of the EQ-5D scale. Logistic regression was used to assess the association between family cohesion and recovery at 3 months.

**Results:**

Of the overall sample, 169 (67.8%) responded to the second interview, and three patients passed away. About 95.2% of patients reported disabilities at baseline, while 88.1% continued to report disabilities after 3 months. Forty patients (16.1%) reported suboptimal family cohesion. Of these patients, 37(94.87%) were in pain, 33(82.5%) reported problems with usual activities, 32(80%) faced problems with self-care, 32 (80%) patients had difficulty in mobility, and 23(57.5%) were depressed. Multivariable regression suggested that patients with suboptimal family cohesion were less likely to recover from disabilities.

**Conclusion:**

The prevalence of any disability 3 months after discharge is striking. This study suggests that health outcomes after blunt trauma are affected by the strength of the patient’s family cohesion. More research is needed to identify effective ways through which the provision of social support can reduce short term disability after trauma.

## Introduction

Globally, injuries are the ninth leading cause of mortality, and it is expected to be the seventh leading cause of death by the year 2030 (Who 2010). Despite this increasing burden, mortality rates of severely injured patients have declined 20% because of improved focus on the treatment of post-injury (Bardenheuer et al., 2000). Consequently, disability rates due to injuries may be increasing and causing a significant burden on population health (Murray et al. 2012). Unlike mortality, comprehensive information on the magnitude and predictors of injury disability is limited. Moreover, health-outcomes following hospital discharge is poorly understood (Richmond et al. 2003).

Disability is a complex interplay of factors, and it extends beyond the physical injury itself; therefore, evaluating risk factors for it is instrumental in preventing long term consequences (Nagi 1991). Prior studies have acknowledged the importance of identifying early predictors and factors associated with disability to facilitate early intervention and improve outcomes (Richmond et al. 2003). Factors that may affect recovery from injuries include age, sex, and type of injury may influence a patient’s prognosis (Richmond et al. 2003). Other factors like social factors can be associated with reduced quality of life (QOL) and a high disability rate (Prang et al. 2015b). Learning about trauma disability and identifying the long-term outcomes of injury is critical to improve patient’s life and to help healthcare professionals provide optimal treatment solutions (Alghnam et al. 2014a).

Saudi Arabia has suffered from significantly high rates of morbidity and mortality due to injuries because they are the second leading cause of death nationwide (Alghnam et al. 2014a). Every year, there are over one million traffic crashes in Saudi Arabia (Alwatan Online, 2015). More importantly, over 86,000 victims died because of crashes in the last two decades (Mansuri et al. 2015). One study suggests that the mortality rate among severe trauma patients can be as high as 8.5% (Alghnam et al. 2014b).

To reduce the burden of injuries, the government started to invest heavily in traffic safety as part of a nationwide initiative known as Saudi vision 2030(Saudi Vision 2030, 2016). One of the most effective preventive measures is the speed camera system, which was implanted in 2010 and was found to reduce mortality and severity of injuries (Alghnam et al. 2017a). Another significant preventive method is the enforcement of road traffic laws such as wearing seatbelt law. One study found that seatbelts use ranged between 4 and 40% among drivers and passengers in Saudi Arabia, which is substantially lower than in developed countries (Bendak 2005).

Although there are studies that examined predictors of trauma death, limited studies, and scarce data have been reported in the literature about the exact burden of nonfatal injuries (Alghnam et al. 2014a). The lack of such vital data limits our understanding of the prevalence of disabilities.

In the Kingdom, family cohesion and social support differ from other cultures due to several factors including religious beliefs, cultural traditions, and reliance on relatives (Countrystudies.us., 2017). Unlike other cultures, Saudis tend to live with their parents until marriage, meaning citizens rarely live alone (Countrystudies.us., 2017). In addition, families tend to be relatively large with an average household size of 6.4 members (Abdul Salam et al. 2014). Islam is the main religion in Saudi Arabia, and in Islam the family is the fundamental block of the society. For example, according to Islamic values, parents are highly respected, marriage is encouraged, and connection with other family members is important (Hamdan, 1990). Traditionally, families in Saudi Arabia are extended families, however, nuclear family structure has been increasing over the last decades (Hamdan, 1990). It is unknown what is the role that family relationships can play to help the patient recover after injuries. It is possible that injured patients can benefit from family support in seeking and complying with rehabilitation programs, which can facilitate an improved outcome (Gabert-Quillen et al. 2012; Nijs et al. 2011). On the other hand, injured patients may increase their reliance on family without a focus on improving their own health. Consequently, this may lead to increased disabilities. Empirical evidence is warranted to better guide future planning and implementation of rehabilitation programs.

The purpose of this study is twofold: 1) to estimate the prevalence of disability 3 months after hospital discharge; 2) to evaluate the impact of family cohesion on recovery from disability after blunt trauma. Because the disabilities due to injuries are increasing, identifying the factors that affect the recovery is quite crucial to guide planning for interventions aimed to improve trauma outcomes in the country.

## Methodology

This is a prospective study of blunt trauma patients who were admitted to King Abdulaziz Medical City (KAMC), a tertiary care facility in Riyadh. KAMC encompasses 1500 beds and provides services to about 600,000 Saudi National Guard employees and their families. It also treats any patient admitted to the emergency department following an injury despite a lack of insurance coverage. Patients were recruited from the KAMC’s trauma registry that records all trauma admissions (Alghnam et al. 2014a). Patients were included in the study if they suffered any blunt, non-penetrating, non-fatal injuries. These included orthopedic injuries, head injuries, and abdominal injuries. The population also included adults who were 18 to 65 years old and hospitalized for at least 1 day. Furthermore, patients had to be conscious, oriented, and able to speak. We excluded patients who were deceased, intubated, unconscious and those in a drug-induced hallucinogenic state. The study team was alerted by the trauma registry staff once any patient meeting the inclusion criteria was admitted. All the patients meeting the criteria between May 2018 and September 2019 were approached and invited to participate, and those who agreed were interviewed by trained coordinators. This study was reviewed and approved by the Institutional Review Board (IRB) at King Abdullah International Medical Research Center (KAIMRC).

One of the aims of the study was to estimate the prevalence of disability among patients who were admitted to the hospital following blunt injury. Based on that, we estimated that the required sample size is 85 patients. This was based on a 32.5% disability rate and the true estimate being within ten percentage points of the true prevalence and a confidence level of 95% (Alghnam et al. 2017b). We anticipated at least a 10% loss of follow up. Two hundred and forty-nine patients participated in the study.

During the first interview, patients were asked about their demographic information, including educational background, occupation, income, and marital status. In addition, two scales were used to evaluate QOL and family cohesion. Both scales were validated and used in previous studies (Abou Abbas and Al Buhairan, 2017; Aburuz et al. 2009;AlBuhairan et al. 2015; EuroQol Research Foundation 2019). EQ-5D-5L disability scale is composed of five dimensions (mobility, self-care, usual activities, pain/discomfort, and anxiety/depression) (EuroQol Research Foundation 2019). Each dimension consists of five levels of severity ranging from no problem, slight, moderate, severe to very severe problem. Also, a scale numbered from 0 to 100 was used to describe and measure patient’s today’s health status (Aburuz et al. 2009). Any patient who reported slight to very severe problems in any of the dimensions was classified as disabled.

A family support scale was used to assess the patient’s family cohesion. It is composed of four questions that measure the strength of the relationship between the patient and his or her mother, father, spouse, and siblings. The responses ranged from “0” very weak to “4” very strong (Abou Abbas and Al Buhairan, 2017; AlBuhairan et al. 2015). Any patient who reported moderate, weak or very weak relationships in any of the questions was considered to have suboptimal family cohesion.

The second interview was conducted via the phone 3 months after the hospital’s discharge using the EQ-5D-5L. The follow-up interview lasted no more than five minutes. Three attempts were made to contact patients. If the patient did not answer by the third time, he or she was classified as a non-responder. Of the 249 patients we contacted, 169 patients responded, 75 did not answer in any of the attempts, three patients passed away, and two patients refused to participate. Responders did not differ from non-responders in age, gender, or baseline self-reported health.

For the recovery analysis, patients were classified into two groups: recovery versus no recovery. Patients who reported a disability at baseline in any of the domains and no disability at 3 months follow up were classified as “recovered.” While those who reported problems at baseline then no change at follow up, meaning there was no improvement were classified as “not recovered.” Additionally, those who reported no problem at baseline and then reported problems at follow-up were classified as “not recovered.”

### Statistical analysis

The data was entered using an online database and then analyzed by STATA 15 for Mac. Descriptive data, such as demographics were represented using percentages, means, and standard deviation (SDs). EQ-5D-5L and family cohesion scale were dichotomized into two levels: disabled versus non-disabled, optimal versus suboptimal family cohesion. Injury mechanisms were classified into: traffic crashes, falls, attempted homicide, and others. Continuous and categorical variables were compared between patients across family cohesion groups using t-test and Chi-square tests, respectively. In addition, we evaluated the prevalence of disability in all domains both at baseline and at 3 months following hospital discharge. A *p*-value of < 0.5 was considered as statistically significant.

Univariate and multivariate logistic regression analyses were constructed to assess the relationship between family cohesion and recovery 3 months after hospital discharge. The regression model was used to identify predictors of recovery in any domain of the EQ-5D-5L scale. Potential predictors included age, gender, and hospital length of stay (LOS). The reference for gender was males, while age and hospital LOS were used as continuous variables. The use of LOS was to adjust for differences in injury mix across the two groups. Results are presented as odds ratios (OR) with 95% confidence intervals.

## Results

**Fig. 1:**
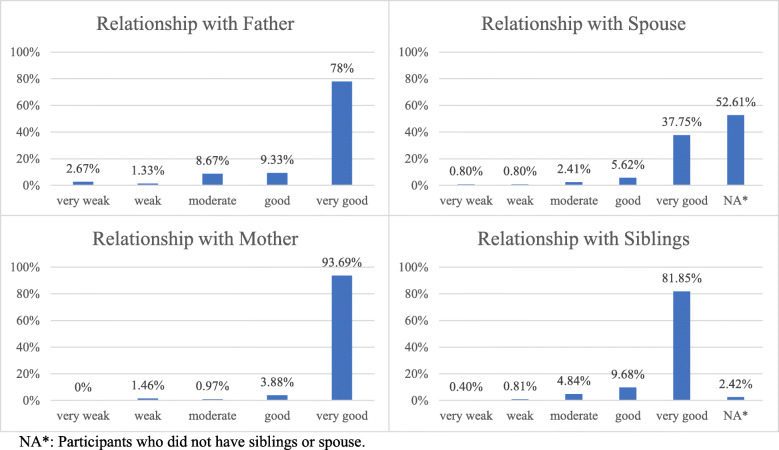
Raw score distribution of family cohesion strength. NA*: Participants who did not have siblings or spouse

We interviewed 249 patients at baseline. Of them, 169 patients were included in the follow-up analysis. The distribution of reported family cohesion scale is shown in Fig. 1. As shown in Table 1, forty patients (16.0%) reported suboptimal family cohesion, while 209 (83.9%) reported optimal family cohesion. Males accounted for over two-thirds of the sample (*n* = 191, 76.7%). Patients who reported low income represented 69.0% (*n* = 172) of the population. The most common mechanism of injury was traffic crashes (*n* = 137, 55.0%). The majority of patients were single (*n* = 134, 53.8%), had a high school degree or less (*n* = 152, 61.04%) and most were employed in governmental sectors (*n* = 126, 50.6%). Patients who reported optimal family cohesion group were slightly older than those in the suboptimal group.

**Table 1:** Descriptive characteristics of the blunt trauma patients

Variable	Family cohesion		***p***-value
	**weak*n*** **= 40**	**strong*n*** **= 209**	
**Age, mean (SD)**	33 (13.80)	35 (13.64)	
**Gender, count (%)**			
Male	28 (70%)	163 (78%)	0.27
Female	12 (30%)	46 (22%)	
**Educational level, count (%)**			
Diploma/ university	10 (25%)	60 (28.71%)	0.33
High school or below	23 (57.5%)	129 (61.72%)	
No education	7 (17.5%)	20 (9.57%)	
**Occupation, count (%)**			
Government	18 (45%)	108 (51.67%)	0.51
Private	2 (5%)	15 (7.18%)	
Student	4 (10%)	27 (12.92%)	
Not working / retired	16 (40%)	59 (28.23%)	
**Income, count (%)**			
High	5 (12.5%)	35 (16.75%)	0.81
Low	29 (72.5%)	143 (68.42%)	
Refused to answer	6 (15%)	31 (14.83%)	
**Marital status, count (%)**			
Married	16 (40%)	98 (47.12%)	0.41
Single	24 (60%)	110 (52.88%)	
**Financial provider, count (%)**			
Yes	15 (37.5%)	94 (44.98%)	0.38
No	25 (62.5%)	115 (55.02%)	
**Cause of injury, count (%)**			
MVC	21 (52.5%)	116 (55.5%)	0.11
Fall	14 (35%)	69 (33.01%)	
Attempted Homicide	3 (7.5%)	3 (1.44%)	
Other	2 (5%)	21 (10.05%)	

Of the patients interviewed at baseline, 237 (94.8%) reported a disability (Table 2). Although patients in the suboptimal family cohesion group reported a lower score on the self-rated health measures than the optimal family cohesion group (respectively 56.67, 61.30), the difference was not statistically significant. Of the overall sample, 165 (66.2%) of patients had difficulty in mobility, 92 (36.9%) were depressed, 214 (86.2%) were in pain, 171 (68.6%) faced problems with self-care, and 183 (73.4%) faced difficulties with usual activities such as driving and house chores.

**Table 2:** Health outcomes at baseline based on family cohesion

Variable	Weak N = 40	Strong N = 209	***p***-value
**ISS**	6.85 (5.3)	7.54 (6)	0.49
**Hospital LOS**	8.87 (8.05)	9.37 (11.07)	0.78
**Health today scale**	56.67 (25.05)	61.30 (24.33)	0.27
**Reported disability**			
Yes	40 (100%)	197 (94.24%)	0.12
No	0 (0%)	12 (5.74%)	
**Difficulty in mobility, count (%)**			
Yes	32 (80%)	133 (63.64%)	0.05
No	8 (20%)	76 (36.36%)	
**Problems in self-care, count (%)**			
Yes	32 (80%)	139 (66.51%)	0.09
No	8 (20%)	70 (33.49%)	
**Problems in usual activity, count (%)**			
Yes	33 (82.5%)	150 (71.77%)	0.16
No	7 (17.5%)	59 (28.23%)	
**Pain/ discomfort, count (%)**			
Yes	37 (94.87%)	177 (84.69%)	0.09
No	2 (5.13%)	32 (15.31%)	
**Depression/ anxiety, count (%)**			
Yes	23 (57.5%)	69 (33.01%)	0.003
No	17 (42.5%)	140 (66.99%)	

Family cohesion was associated with disability. We found that patients with suboptimal.

family cohesion reported more disability in terms of pain, mobility, self-care, and inability to perform usual activities. Difficulty in mobility and depression and anxiety significantly higher among those with suboptimal family cohesion (*P* < 0.05).

**Fig. 2:**
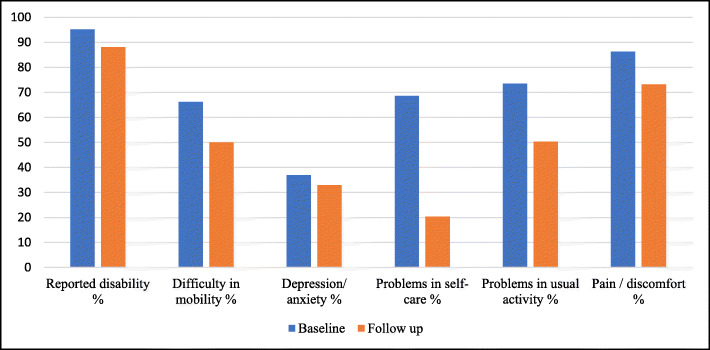
The prevalence of any disability in EQ-5D at baseline and at 3 months

As shown in Fig. 2, the follow-up interview showed a minimal recovery where 88.1% of patients continued to report any disability at 3 months. Furthermore, close to three-quarters of the sample reported pain or discomfort, 84 (50%) reported difficulty in mobility, 54 (32.1%) were depressed, and 84 (50.3%) could not perform a usual activity. The most improvement was in the self-care domain, where only 34 (20.3%) reported any problems.

The regression analysis identified the optimal family cohesion as a significant predictor of health outcomes 3 months after discharge (Table 3). Patients with suboptimal family cohesion were 75% less likely to recover from depression (OR 0.25 95% CI: 0.1–0.6), 69% less likely to recover in mobility (OR 0.31 95% CI 0.11–0.82). A multivariable logistic regression analysis was constructed to adjust for potential confounders (age, gender, and hospital LOS). Patients with suboptimal family cohesion were 77% (OR 23 95% CI 0.09–0.6) less likely to recover from depression (OR 23 95% CI 0.09–0.6) and 73% less likely to recover in the mobility domain (OR 0.27 95% CI 0.1–0.77) regardless of age, gender, and hospital LOS.

**Table 3:** Logistic regression model of the association of family cohesion and reported disability after three months

	Univariate			Multivariate		
**Depression**						
**Variable**	**OR**	**95% CI**	***P*-value**	**OR**	**95% CI**	***P-*value**
**Age**				0.99	0.96–1.01	0.33
**Gender**				1.2	0.52–2.8	0.67
**Hospital LOS**				1.01	0.97–1.05	0.62
**Suboptimal Family Cohesion**	0.25	0.1–0.6	0.003	0.23	0.09–0.6	0.002
**Usual Activity**						
**Age**				1	0.97–1.02	0.9
**Gender**				0.5	0.22–1.1	0.08
**Hospital LOS**				0.97	0.93–1.01	0.13
**Suboptimal Family Cohesion**	0.4	0.15–1.02	0.06	0.44	0.17–1.16	0.1
**Self-care**						
**Age**				0.96	0.96–1.01	0.33
**Gender**				0.6	0.25–1.45	0.255
**Hospital LOS**				0.96	0.92–1	0.03
**Suboptimal Family Cohesion**	0.93	0.31–2.67	0.9	0.99	0.33–3	0.99
**Mobility**						
**Age**				0.97	0.95–1	0.03
**Gender**				1.11	0.5–2.48	0.8
**Hospital LOS**				0.95	0.91–0.99	0.02
**Suboptimal Family Cohesion**	0.31	0.11–0.82	0.02	0.27	0.1–0.77	0.01
**Pain**						
**Age**				1.01	0.99–1	0.32
**Gender**				0.31	0.11–0.92	0.03
**Hospital LOS**				0.91	0.85–0.96	0.02
**Suboptimal Family Cohesion**	0.75	0.26–2.15	0.6	0.93	0.31–2.8	0.91

## Discussion

Our study found that most blunt trauma patients continued to report disability 3 months after hospital discharge. This finding highlights the significant burden disability pose due to injuries on population health of the Kingdom. The prevalence of disability presented here is higher than the findings of other international studies. For example, Kalahroudi et al. found that 55.8% reported disability at 3 months follow up (Abedzadeh-Kalahroudi et al. 2015). This discrepancy may be due to the differences between the two populations or differences in the instrument to capture disability. A similar finding by holtslag et al. was also reported in the five EQ-5D-5L dimensions of mobility (48%), self-care (18%), daily activities (55%), pain and discomfort (63%), and anxiety or depression (28%) for patients following trauma (Holtslag et al. 2007). It is noteworthy to state that their follow up period was long (between 12 and 18 months). It was not possible to compare our findings to local studies since none of the previous literature in Saudi Arabia followed patients longitudinally.

The purpose of this study is to determine the impact of family cohesion on the quality of life and disability of patients after blunt trauma. Patients who reported suboptimal family cohesion were less likely to recover in the mobility domain. These results are consistent with Prang et al., where they found that having strong support from family and friends had a positive impact on physical improvement and return to optimal function after road traffic injury (Prang et al. 2015a). Additionally, Harms et al. reported that having strong family support could affect recovery from injury and reduce the number of related complications (Harms and Talbot 2007). However, our results differ from those of Richmond et.al*,* where they reported that availability of social support at the time of injury did not reduce disability (Richmond et al. 2003). A possible explanation for this might be the difference in the assessment tools, follow up time, and cultural background.

As stated earlier, family structure in Saudi Arabia may increase reliance on relatives due to several factors, including religious beliefs, large family size, and cultural views (Abdul Salam et al. 2014). In the US, one study showed that patients who reported low social support,which includes family support, after 1 year of traumatic injuries had higher odds of poor psychological outcomes which negatively impacted their recovery (Carr et al. 2020). However, other studies suggested that family cohesion could have a negative impact by increasing reliance on others and decreasing independence (Turk et al. 1992). More research is needed not only to identify the link between the presence of family support and enhanced resilience after blunt injury but also effective ways through which the provision of social support can enable achieving positive health outcomes.

Our study suggests that patients who have suboptimal family cohesion reported higher depression and anxiety at baseline. After 3 months, depression and anxiety were still significant disabilities among participants who lacked family cohesion. The relationship between family cohesion and mental health is well established in prior literature (Birkeland et al. 2017; Thoits 2011; Yaşan et al. 2009. Guest et al. showed that psychological stress had been an essential factor in the recovery of musculoskeletal injuries, and psychological stress has strong ties with a lack of family cohesion (Guest et al. 2017). In addition, Coronas et al. found that family cohesion is associated with the onset of post-traumatic stress disorders after road traffic injuries (Coronas et al. 2008). The absence of appropriate family cohesion may lead to depression, anxiety. Jansen et al. suggest that having optimal family cohesion plays a central role in processing traumatic experiences (Janssen et al. 2008). This is in line with the evidence presented by Charuvastra et al., who reports that the presence of family cohesion results in better emotional control, which in turn diminishes the likelihood of developing post-traumatic stress disorders (Charuvastra and Cloitre 2008). Studies showed that the availability of a social network reduced both physical and psychological disability (Hilari et al. 2010; Mortimore et al. 2008; Symonette et al. 2013). These findings may point to the need for early detection and intervention by healthcare personnel, social workers in particular.

The findings of this study have several important implications for future practice. The high prevalence of disability should guide policymakers to evaluate whether more rehabilitation centers are needed in order to reduce the disability burden in the country. Rehabilitation centers can provide patients with comprehensive recovery programs that focus on improving physical, social, and mental factors. Additionally, this study highlights the need to identify patients with suboptimal family cohesion in order to offer early intervention and reduce the likelihood of short-term disability. These include health home services, psychological counseling, and support groups. Understanding the social determinants of health is a critical step in clinical practice. We suggest that healthcare providers and social workers collaborate to develop interventions that strengthen social support.

There are several limitations to this study that need to be acknowledged. First, this study included a relatively small sample size, which may have affected the power to evaluate all the factors associated with recovery. Only 169 patients out of 249 were reached in the second interview. Those who did not respond could have had worse disability levels, which may underestimate the level of disability presented in this study. Second, the generalizability of these results is limited because it was conducted in a single trauma center. It is also important to highlight that KAMC is one of the few hospitals that provide advanced trauma care nationwide. Thus, we might underestimate the prevalence of disability in the country. Third, the follow-up period was limited to 3 months; therefore, long term disability and recovery were not evaluated. Scarce data exist on the ideal follow-up timeframe of trauma patients. Most trauma survivors appear to recover reasonably quickly as within the first 3 months, which is why we chose this follow up timeframe (Mitchell et al. 2012). Longer follow up may provide additional insights into the prevalence of long-term disabilities due to injuries in Saudi Arabia.

Fourth, our study did not capture preexisting conditions. Though, our patient population was relatively young, and it is reasonable to assume the prevalence of preexisting conditions and baseline impairment were minimal and that it did not differ between the two groups. Fifth, other relevant factors that could potentially affect the patient’s ability to cope after trauma, such as environmental factors, were not assessed. Sixth, family cohesion was not reassessed after 3 months. Despite limitations, this study has several strengths. It is a prospective study that used a well-validated health status scale to measure QOL with a standardized timeframe of the outcome assessment. To our best knowledge, no previous study has investigated the association of family cohesion on quality of life after trauma in Saudi Arabia.

## Conclusion

Our findings suggest that family cohesion is associated with the disability following blunt injuries. Implementing methods to strengthen family cohesion via rehabilitation centers may decrease the rate of disability after blunt injuries. Increasing awareness of the importance of family cohesion in post-trauma care specifically plays a fundamental role in strengthening it. Also, family counseling services should be offered as a part of post-trauma care in every hospital. Social workers are recommended to work in partnership with health workers to enhance patient quality of life. More investment is needed to reduce the frequency and severity of traffic crashes in order to reduce their consequences on population health.

## Data Availability

The data that support the findings of this study is available upon reasonable request from Dr. Alghnam, S.

## Abbreviations

QOL: Quality of Life
LOS: Length of Stay
OR: Odds Ratio
CI: Confidence Interval
